# Herbivory Drives the Spread of Salt Marsh Die-Off

**DOI:** 10.1371/journal.pone.0092916

**Published:** 2014-03-20

**Authors:** Mark D. Bertness, Caitlin P. Brisson, Matthew C. Bevil, Sinead M. Crotty

**Affiliations:** Department of Ecology and Evolutionary Biology, Brown University, Providence, Rhode Island, United States of America; North Carolina State University, United States of America

## Abstract

Salt marsh die-off is a Western Atlantic conservation problem that has recently spread into Narragansett Bay, Rhode Island, USA. It has been hypothesized to be driven by: 1) eutrophication decreasing plant investment into belowground biomass causing plant collapse, 2) boat wakes eroding creek banks, 3) pollution or disease affecting plant health, 4) substrate hardness controlling herbivorous crab distributions and 5) trophic dysfunction releasing herbivorous crabs from predator control. To distinguish between these hypotheses we quantified these variables at 14 Narragansett Bay salt marshes where die-off intensity ranged from <5% to nearly 98%. Nitrogen availability, wave intensity and plant growth did not explain any variation in die-off. Herbivory explained 73% of inter-site variation in die-off and predator control of herbivores and substrate hardness also varied significantly with die-off. This suggests that salt marsh die-off is being largely driven by intense herbivory via the release of herbivorous crabs from predator control. Our results and those from other marsh systems suggest that consumer control may not simply be a factor to consider in marsh conservation, but with widespread predator depletion impacting near shore habitats globally, trophic dysfunction and runaway consumption may be the largest and most urgent management challenge for salt marsh conservation.

## Introduction

Salt marsh die-off is a growing phenomenon in the western Atlantic [1] and a serious conservation problem as salt marshes provide valuable ecosystem services to coastal habitats [2]. Landscape wide die-offs were first described over 35 years ago in Hudson Bay, Canada, where increasing geese populations, fueled by artificial nitrogen fertilizer use, led to runaway grazing and marsh collapse [3]. Nearly 15 years later, salt marsh die-offs were described on the Southeastern and Gulf coasts of the U.S. driven by snail herbivory and exacerbated by drought, which increased the vulnerability of marsh cordgrass to consumers and fungal infections [4]. Most recently, salt marsh creek bank die-offs of the cordgrass *Spartina alterniflora* are occurring in New England, driven by predator depletion and release of the herbivorous marsh crab *Sesarma reticulatum* (hereafter *Sesarma*) from consumer control [5]. Historical reconstructions have revealed that Cape Cod die-offs have been ongoing, unnoticed for over two decades as a consequence of human population growth and recreational fishing pressure depleting predator populations [6], [ 7].

While it has been hypothesized that human impacts causing trophic dysfunction are driving these die-offs, salt marshes are widely considered to be controlled exclusively by physical forces [8], [ 9]. Consequently, many recent studies and reviews of salt marsh die-off fail to even acknowledge the impact of predator depletion and herbivory on die-off [10]–[13]. For example, a recent study on Cape Cod proposed from studies at a single site that eutrophication was causing salt marsh die-offs worldwide [10]. Other hypothesized causes of marsh die-off include harsh physical conditions e.g. hypersaline and anoxic soils [13], fungal infections [14], boat wakes [15], pollution [15], substrate type [16] and positive interactions between eutrophication and increased herbivory [17].

Recently, creek bank salt marsh die-off has spread into Narragansett Bay, where it had been reported absent as recently as 2009 [5]. We use this spread of salt marsh die-off to examine the relative importance of proposed drivers of die-off. We studied 14 Narragansett Bay salt marshes with a broad range of die-off conditions. We used archived aerial photographs to quantify the temporal pattern of die-off and then tested the competing hypotheses listed above with field measurements. To distinguish between hypotheses we quantified die-off, nitrogen availability (eutrophication), common source transplant performance (pollution and disease), wave exposure (boat wakes), herbivory, substrate hardness and predation on herbivorous crabs at each site (trophic dysfunction). We then used multiple regression to quantify the contribution of each of these factors to the spread of die-off.

### Ethics Statement

All necessary permits for field sampling were obtained from the Cape Cod National Seashore.

## Materials and Methods

### Extent and Time Course of Die-Off

Fourteen accessible salt marshes with a range of die-off conditions were identified in Narragansett Bay. The extent of die-off was measured at each site in July 2013 by walking two randomly placed 100 m creek bank transects and every 10 m measuring the total width of the cordgrass zone and the width of the cordgrass zone denuded by die-off.

We quantified the history of die-off at each site using archived aerial photographs from 1997, 2003, 2008, and 2012. Marsh habitat was defined as the total area covered in visible salt marsh vegetation and die-off area was defined as the total area converted from marsh vegetation to barren peat over each time interval. All areas were defined and quantified using ArcGIS. Marsh linear extent was defined as the total length of marsh edge bordering water (length of marsh border exposed to die-off conditions). To test the hypothesis that salt marsh die-off in Narragansett Bay is recent, percent linear extent affected by die-off was calculated for each site and time point. We tested the hypothesis that the intensity of die-off is increasing over time by measuring the mean width of die-off and the mean rate of die-off expansion for each site and time period. Mean die-off width was calculated as total area of die-off divided by length of border affected. Rate of die-off expansion was calculated as mean die-off width per year. An ANOVA with a univariate split plot approach was used to analyze both percent linear extent and the width of die-off over time.

### Plant Growth Potential

In May 2013, an undamaged common source core (7 cm diameter) of short-form *Spartina alterniflora* (hereafter cordgrass) was transplanted to the creek bank of each site. It was protected from crab, bird and mammal herbivory by a 1 cm mesh hardware cloth top-less cylindrical cage that extended 20 cm belowground and 30 cm aboveground, a height tall enough to exclude any herbivores. Mesh size was chosen to allow ample sunlight and room for cordgrass growth. Cage controls were not necessary since extensive previous work on Cape Cod and Long Island Sound showed no caging effects on growth using this technique [5]–[7]. Aboveground biomass was harvested in mid August after flowering, dried to a constant weight and weighed. Aboveground cordgrass biomass/core from caged cores was used as a measure of site quality for plant growth, to detect inter-site variation in pollution [15] or disease [14] affecting plant growth or mortality.

### Eutrophication

To examine the hypothesis that eutrophication contributes to inter-site variation in creek bank die-off [10], we quantified inter-site differences in cordgrass leaf nitrogen. Ambient nitrogen levels limit cordgrass production and cordgrass responds to increased nitrogen availability by increasing aboveground growth as well as tissue nitrogen levels [18]. Therefore, we used cordgrass leaf percent nitrogen as a site-specific indicator of nitrogen availability. Leaf tissue from each common source core was harvested after a growing season, oven dried, ground in a Wiley Mill, dried at 60 °C before further grinding and analyzed for percent nitrogen with a ThermoQuest CE Instruments Model NC2100 elemental analyzer.

### Wave Exposure

To examine the hypothesis that boat wakes or wave exposure contributed to creek bank die-off via erosion [15] we deployed magnesium calcite chalk blocks (n  =  10/site) [19] on the creek bank at all 14 sites in July 2013. Cylindrical pre-weighed (5×2 cm, DXH) chalk blocks, sealed on the side with polyurethane were glued to hardware cloth bases with a polyphenol adhesive. They were pinned to the substrate with wire staples and left in the field for five weeks and then dried and reweighed. Chalk block dissolution was used as a time integrated site measure of wave and wake exposure.

### Herbivory

*Sesarma* herbivory was measured at each site the second week of July 2013. Herbivory was estimated by walking a 20 m transect along the creek bank of each site and every 2 m scoring 100 cordgrass stems for crab herbivore damage in a 50×50 cm quadrat. *Sesarma* grazing leaves characteristic rasped edges and clipped blades on transplanted cordgrass [5].

### Substrate Hardness

Substrate hardness, an accurate proxy for peat density [20], was measured with a penetrometer in vegetated areas along the grazing border at all sites. The penetrometer was a top loading spring scale with a 2 cm diameter, 7 cm long rod mounted vertically on the weighing platform. The force required to push the rod vertically into the substrate (n = 10/site) was recorded avoiding burrows along the same 20 m transect line used to assess crab herbivory.

### Predation Pressure on Sesarma

To quantify trophic dysfunction in Narragansett Bay, operationally defined as how severely predator depletion has reduced predation pressure on *Sesarma* leading to increased *Sesarma* densities and herbivory, we quantified *Sesarma* densities, predation pressure on tethered *Sesarma*, and top predator biomass at each study site.

*Sesarma* abundance was estimated with nocturnal pitfall traps in late July when the crabs showed maximum activity (n  =  8 traps/site) [6]. *Sesarma* were counted and measured the following morning. *Sesarma* tethering assessed inter-site variation in predation pressure. *Sesarma* were tethered to braided monofilament fishing line, secured with cyanoacrylic glue, and fastened using metal pins in *Sesarma* habitat at each site (n  =  15 *Sesarma*/site). Caged crabs to assess tethering artifacts were not deployed because previous studies on Cape Cod and Long Island Sound found no tethering artifacts [5], [6]. Tethered *Sesarma* behave normally on tethers, digging shallow burrows and foraging in the tethered area. Crabs were deployed overnight in August 2013 and mortality was scored the following morning. Predation mortality left broken carapace fragments attached to tethers, mortality from desiccation left dead intact crabs, while surviving tethered crabs were found in newly excavated shallow burrows.

Inter-site variation in predator populations was quantified with baited, commercial 30×60×90 cm traps with funnel openings (n  =  3 traps/site) in August 2013. Traps were constructed of 2.5 cm PVC-coated steel mesh and deployed overnight in creeks >100 m apart. Traps have been shown to catch dominant *Sesarma* predators in the past and thus provide reliable estimates of predatory populations [6]. All individuals caught were identified to species, measured (carapace width for crabs, snout-vent length for finfish, total length for eels) and released alive. Biomass was estimated using species- and sex-specific scaling equations [21]–[23] and pooled for each site.

Multiple regression with leaf tissue percent nitrogen, cordgrass transplant biomass, chalk block dissolution and herbivore intensity was used to examine alternative hypotheses explaining inter-site variation die-off among sites. To examine the hypotheses that site differences in herbivory were being driven by inter-site differences in top predator abundance and predation on *Sesarma* [6], [7] and/or that substrate hardness limits *Sesarma* burrowing and herbivory, we also performed linear regressions between predator biomass, predation rates on Sesarma, substrate hardness and herbivory. All variables were transformed as needed when necessary. Data was analyzed in JMP 10.

## Results

**Figure 1:**
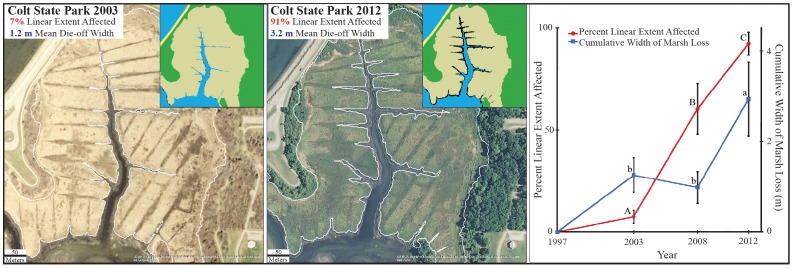
Historical reconstruction of study sites. Images and analysis of study site aerial photographs illustrate the progression from a healthy state in 2003 (Left) to a declining state with high levels of die-off in 2012 (Center). Please note that the color differences between the pictures are due to differences in picture quality between 2003 and 2012. Take note of the white line tracing die-off in the main pictures and the highlighted black area denoting die-off in the pictures in the upper right hand corners. Percent linear extent and cumulative width of marsh lost to die-off rapidly accelerated at Narragansett Bay salt marshes over the past decade (Right). Letters indicate significant differences in variables among time intervals (ANOVA, Tukey HSD).

Inter-site variation in die-off intensity ranged from <5% to nearly 98% from ground surveys in 2013. GIS analysis revealed that creek bank die-off (percent linear extent of die-off of available habitat) significantly increased over time (F_2,12_  =  394.02, P < 0.0001). Die-off was absent in Narragansett Bay in 1997, minimal (7%) in 2003, but by 2012 affected >85% of creek bank habitats (Fig 1). Paralleling the linear spread of die-off, the amount of marsh area also damaged by die-off increased over time (F_2,39_  =  50.13, P < 0.0001) nearly 3-fold between 2008 and 2012 (Fig 1).

**Figure 2:**
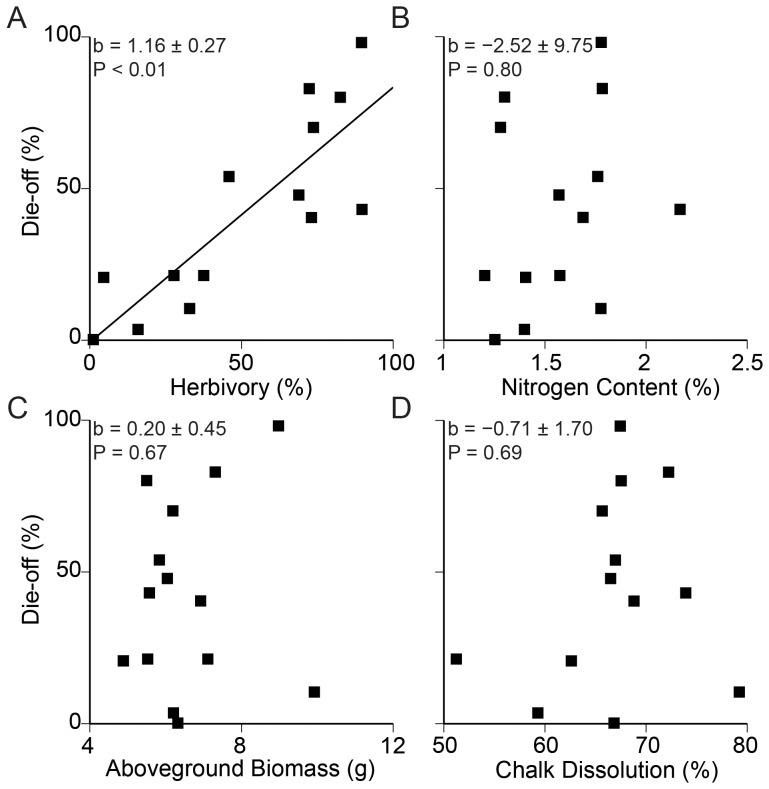
Relationship between herbivory, eutrophication, pollution/disease and wave exposure on site variation in die-off. Multiple regression on the impact of herbivory (A), eutrophication (B), pollution and/or disease (C), and wave exposure (D) on salt marsh die-off. Multiple regression coefficients, standard errors and p values are shown in each graph. Only herbivory (A) significantly contributed to die-off.

Multiple regression between leaf tissue percent nitrogen, aboveground cordgrass transplant biomass, percent chalk block dissolution, herbivore intensity and extent of die-off revealed that inter-site variation in herbivory explained 73% of the inter-site variation in salt marsh creek bank die-off (F_1,8_  =  18.49, P < 0.01, Fig 2A), while nitrogen availability, growth potential and wave exposure did not significantly explain any residual inter-site variation in salt marsh die-off (F_1,8_  =  0.07, P>0.50, Fig 2B; F_1,8_  =  0.20, P>0.50, Fig 2C; F_1,9_  =  0.17, P>0.50, Fig 2D, respectively).

**Figure 3:**
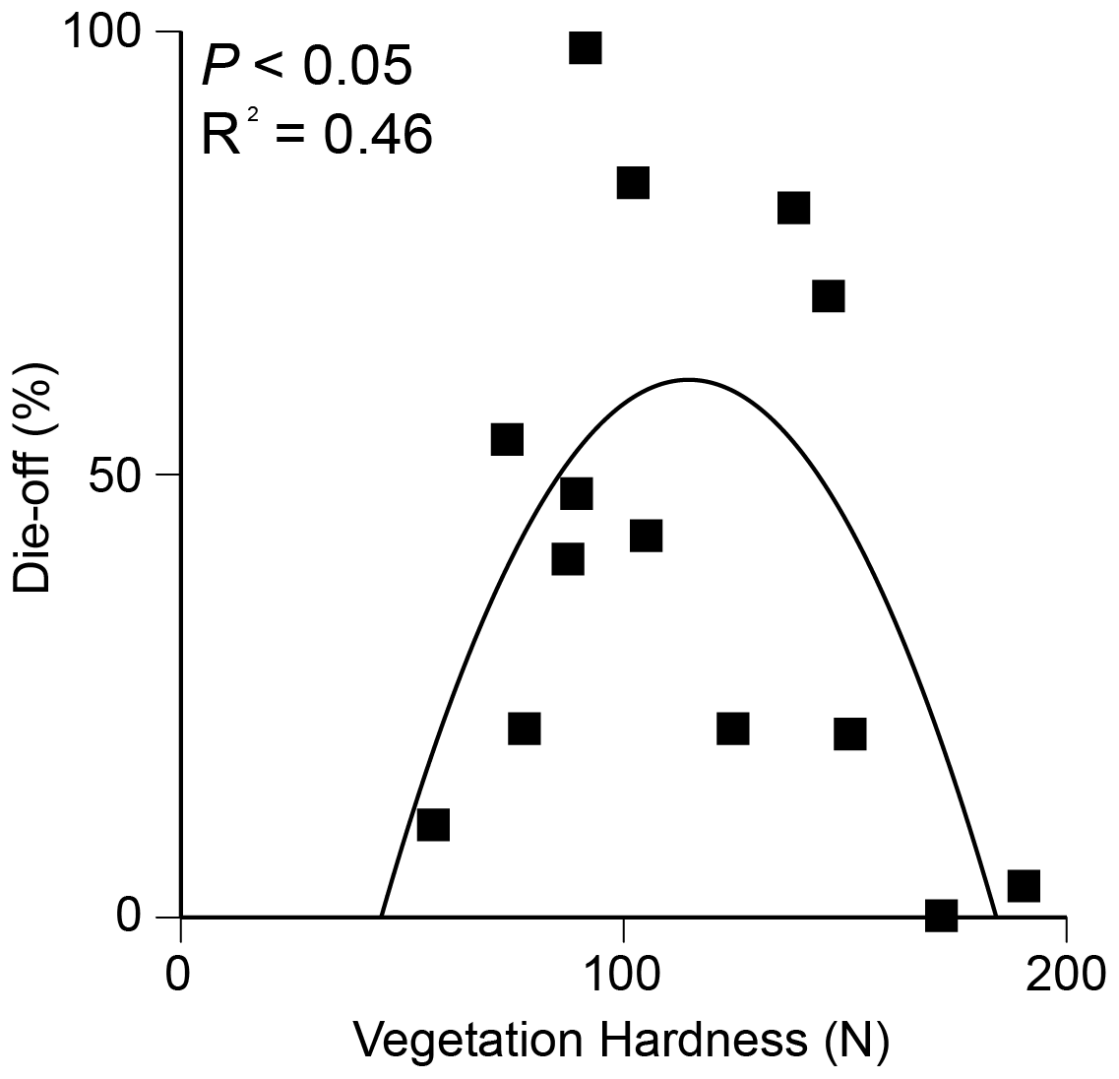
Nonlinear polynomial regression relationship between substrate hardness and die-off. Substrate hardness explained 46% of inter-site variation in die-off. Intermediate substrate hardness experiences the highest level of die-off. Soft substrate cannot support burrows while hard substrate is too hard to burrow in.

We performed a separate polynomial regression between substrate hardness (N) and herbivory to test the hypothesis that substrate hardness dictates crab herbivory. Variation in substrate hardness in vegetated areas along the grazing border explained 46% of variation in salt marsh creek bank die-off (F_2,11_  =  4.63, P < 0.05, Fig 3).

**Figure 4:**
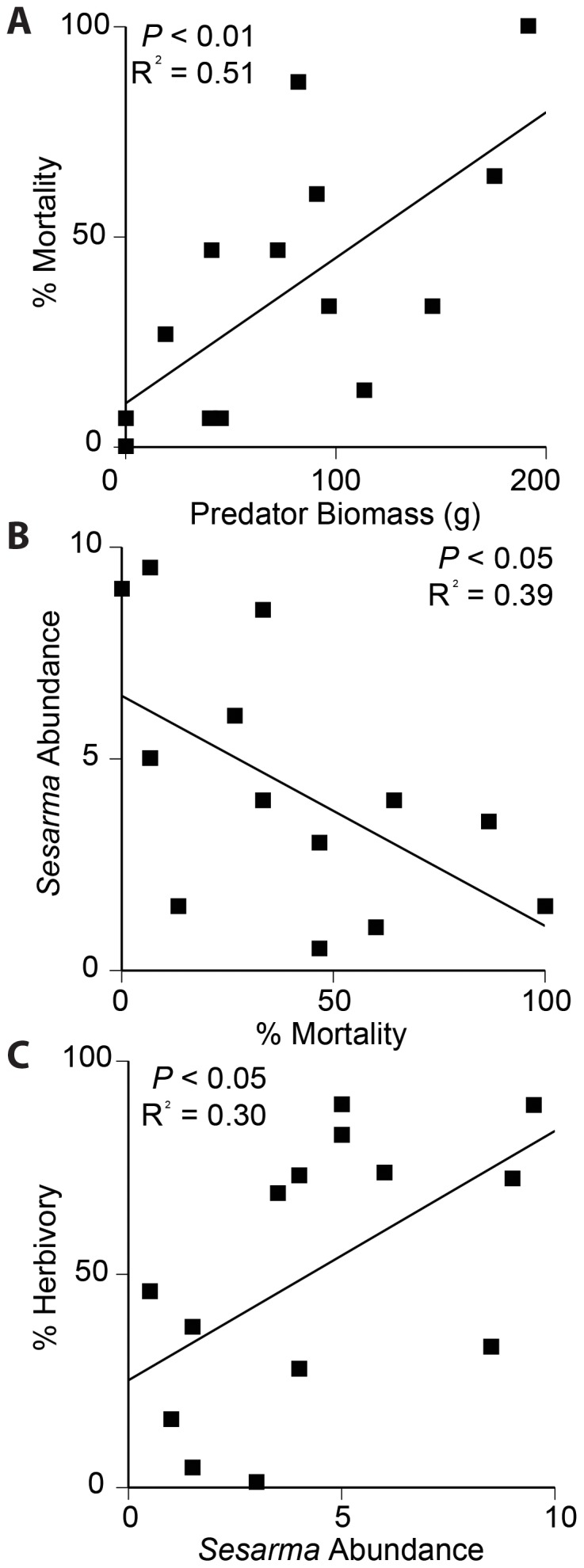
Trophic dysfunction and salt marsh die-off. As predator biomass decreases, predation mortality decreases (A), increasing *Sesarma* abundance (B), and leading to increased herbivory (C).

To test the hypothesis that creek bank die-off is caused by trophic dysfunction due to overfishing, we performed regressions across trophic levels (Fig 4) to test if the previously established trophic cascade in Cape Cod and Long Island Sound marshes had spread to Narragansett Bay [6], [7]. We found a positive relationship between predator abundance and predation pressure; predator abundance explained 51% of the inter-site variation in predation pressure (F_1,12_  =  12.55, P < 0.01, Fig 4A). Predation pressure was negatively associated with *Sesarma* abundance, explaining 39% of inter-site variation in *Sesarma* abundance (F_1,12_  =  7.81, P < 0.05, Fig 4B). *Sesarma* abundance was positively correlated with herbivory, explaining 30% of the inter-site variation in herbivory across sites (F_1,12_  =  5.30, P < 0.05, Fig 4C).

## Discussion

Our results reveal that the spread of creek bank die-off into Narragansett Bay salt marshes is being driven by predator depletion, which releases herbivorous crabs from consumer control. Additionally, salt marshes with medium substrate hardness are the most vulnerable to die-off. Our results reject the alternate hypotheses that eutrophication, boat wakes, plant disease and/or pollution are associated with the spread of salt marsh die-off into Narragansett Bay and suggest that they are not drivers of the widespread die-off of western Atlantic salt marshes.

### Causation Based on Correlations

While many explanations have been proposed for salt marsh die-off, few have been rigorously tested by well-replicated field experiments. We found no evidence that eutrophication (Fig 2B), pollution or disease affecting plant growing conditions (Fig 2C), or wave exposure (Fig 2D) are directly influencing the spread of salt marsh die-off. None of these proposed die-off mechanisms have been tested experimentally at multiple sites. Based on studies at a single site, Deegan et al. (2012) proposed that eutrophication leads to decreased belowground cordgrass investment, decreasing cordgrass biomass and triggering global salt marsh creek bank collapse. In an identical experiment was independently replicated in Long Island Sound, no shift in biomass allocation or creek bank collapse was found in a 5-year time span [24]. In addition, at Sippiwissett Marsh on Cape Cod, >30 years of nitrogen additions have not led to creek bank collapse [25] and at our Cape Cod study sites we have never seen creek bank die-off without evidence of extensive *Sesarma* burrowing which increases vulnerability to calving and marsh loss [6], [7].

Of all of the hypotheses proposed to cause die-off, herbivory remains the only one that has been widely examined experimentally in well-replicated studies across the Canadian subarctic [3], New England [6], [7], Southeastern and Gulf coasts of the United States [4], and the east coast of South America [26]. Our study adds further support to the hypothesis that herbivory is the central driving mechanism of salt-marsh die off. Additionally, our study reveals that herbivory may be mediated by substrate hardness. Substrate hardness determines where runaway herbivory can occur because it limits crab burrowing in hard and soft substrates, leading to peak herbivory in medium hardness substrate creek banks, where burrows can be easily constructed and maintained [16].

Historically, salt marsh ecosystems have been thought to be controlled by physical forces based on correlations between marsh productivity and physical factors [8], [9]. Globally, however, near shore food webs have been severely altered by overfishing and predator depletion has heavily impacted seagrass beds [27], kelp forests [28] and coral reefs [29]. Here we have shown that salt marsh ecosystems are also vulnerable to consumer control and consumer mediated salt marsh die-offs are an emerging phenomenon that needs to be addressed.

### Management Implications

Our results predict a dire future for New England salt marshes. Salt marshes are one of the most valuable ecosystems on earth per unit area because they provide coastal storm buffering, biochemical processing, carbon sequestration, and nursery habitat ecosystem services [5]. With increasing human population growth in coastal areas, commercial and recreational fishing pressure and predator depletion will inevitably increase [30]. Globally, all salt marsh conservation and management is based on the premise that physical factors exclusively control salt marshes. Our results and those from other marsh systems suggest that consumer control may not simply be a factor to consider in marsh conservation, but with widespread predator depletion impacting near shore habitats globally, trophic dysfunction and runaway consumption may be the largest and most urgent management challenge for salt marsh conservation. Rebuilding near shore predatory fish stocks [31] and limiting recreational fishing to catch and release may be the most critical salt marsh conservation measures in saving salt marshes from the impending threat of sea level rise.
